# NO-Releasing Enmein-Type Diterpenoid Derivatives with Selective Antiproliferative Activity and Effects on Apoptosis-Related Proteins

**DOI:** 10.3390/molecules21091193

**Published:** 2016-09-08

**Authors:** Dahong Li, Xu Hu, Tong Han, Jie Liao, Wei Xiao, Shengtao Xu, Zhanlin Li, Zhenzhong Wang, Huiming Hua, Jinyi Xu

**Affiliations:** 1 Key Laboratory of Structure-Based Drug Design and Discovery, Ministry of Education, School of Traditional Chinese Materia Medica, Shenyang Pharmaceutical University, Shenyang 110016, China; lidahong0203@163.com (D.L.); huxu105@163.com (X.H.); hantong1221@163.com (T.H.); howlris@gmail.com (J.L.); lzl1030@hotmail.com (Z.L.); 2 State Key Laboratory of New-Tech for Chinese Medicine Pharmaceutical Processes, National Post-Doctoral Research Workstation, Jiangsu Kanion Pharmaceutical Co. Ltd., Lianyungang 222001, China; kanionwzz2016@126.com; 3 State Key Laboratory of Natural Medicines, Department of Medicinal Chemistry, China Pharmaceutical University, Nanjing 210009, China; cpuxst@163.com

**Keywords:** oridonin, NO donor, antiproliferative activity, diterpenoid, apoptosis related proteins

## Abstract

A series of nine enmein-type *ent*-kaurane diterpenoid and furoxan-based nitric oxide (NO) donor hybrids (**10a**–**i**) were designed and synthesized from commercially available oridonin (**1**). These hybrids were evaluated for their antiproliferative activity against Bel-7402, K562, MGC-803, and CaEs-17 human cancer cell lines and L-02 normal liver cells. The antiproliferative activity against tumor cells was stronger than the lead compound **1** and parent molecule **9** in most cases. Especially, compound **10f** showed the strongest activity against human hepatocarcinoma Bel-7402 cell line with an IC_50_ of 0.81 μM and could also release 33.7 μmol/L NO at the time point of 60 min. Compounds **10a**–**i** also showed cytotoxic selectivity between tumor and normal liver cells with IC_50_ ranging from 22.1 to 33.9 μM. Furthermore, the apoptotic properties on Bel-7402 cells revealed that **10f** could induce S phase cell cycle arrest and apoptosis at low micromolar concentrations. The effects of **10f** on apoptosis-related proteins were also investigated. The potent antiproliferative activities and mechanistic studies warrant further preclinical investigations.

## 1. Introduction

**Figure 1:**
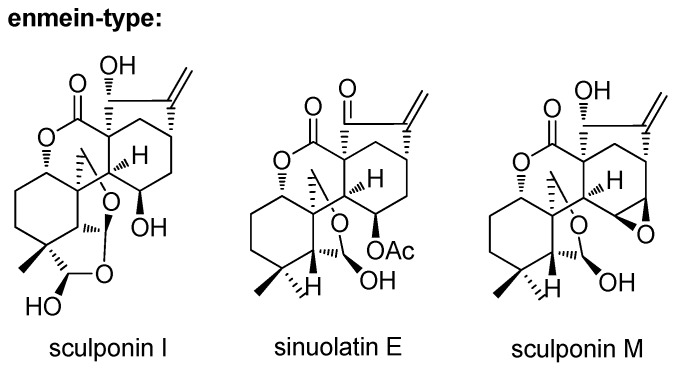
Natural enmein-type *ent*-kaurane diterpenoids.

Natural products and natural product structures continue to play a highly significant role in the drug discovery and development process, especially in the case of antitumor drugs [1]. Diterpenoids, especially *ent*-kaurane diterpenoids, are well known to produce bioactive molecules [2]. Among these, enmein-type 6,7-*seco*-*ent*-kaurane diterpenoids have unique chemical skeletons and exhibit significant activities (Figure 1) [3,4,5]. These natural enmein-type *ent*-kaurane diterpenoids with a lot of stereogenic centers and complex ring systems have attracted much attention [6,7,8,9,10,11,12,13,14,15]. Natural sources cannot afford large compound supply for structure modification. We have found a convenient and feasible method to construct the skeleton of enmein-type 6,7-*seco*-*ent*-kaurane diterpenoid from natural oridonin [9]. Further investigation in the field of medicinal chemistry is still in urgent need.

Nitric oxide (NO), one of the simplest, odorless, colorless, highly reactive, and biological molecules in nature, is a free radical and a key mediator involved in diverse physiological and pathological processes [16,17,18,19,20]. NO-donating compounds have recently come into focus for the treatment of tumors because NO plays a central role in cell regulatory pathways and is a key signaling molecule involved in the death and apoptosis of tumor cells [21,22,23,24,25,26,27,28,29,30,31,32]. Generally, high levels of NO (above 400 nM [33]) produced from NO donors can induce apoptosis, inhibit metastasis of tumor cells, and sensitize tumor cells to radiation, immunotherapy, and chemotherapy. Some synthesized NO-releasing compounds have already shown antitumor activity against human carcinoma cells in vitro and in vivo [21,22,23,24,25,26,27,28,29,30,31,32]. Due to the highly reactive nature of NO, it is difficult to predict its biological effects on a given system from single doses even if provided by the longer action of inducible nitric oxide synthase (iNOS). High concentrations of NO can be generated not only by iNOS, stimulated by cytokines, but also effectively by NO donors. Thus, NO donors, capable of producing a sustained release with a wide range of half-time lives and predictable estimated doses, have become useful tools to study the biological properties of NO in cells and in vivo models of carcinogenesis [34]. Furoxans are stable and represent an important class of NO donors, which can produce high levels of NO in vitro and inhibit the growth of tumors in vivo. The furoxan ring releases NO with the action of thiol cofactors [23]. It is hypothesized that furoxan-ring opening to give a nitroso derivative is accomplished by thiolate attacking position 3 or 4. The derivative then releases NO^−^, which is oxidized to release NO [35,36,37]. The ester molecules characterized by a furoxan-based NO-releasing moiety have already been explored by several research groups for improving the pharmacological profile of the parent drugs [26,38,39,40]. For example, Chen et al. synthesized a series of furoxan-based NO-releasing oleanolic acid derivatives [41]. Six derivatives exhibited strong antiproliferative activity in vitro and released high levels of NO, and two of them showed significant inhibitory activity of human hepatocellular carcinoma tumor in vivo. The hybrids with glycyrrhetinic acid were achieved by Lai et al. [42]. Five derivatives showed small IC_50_ values against human hepatocellular carcinoma tumor cells (0.25–1.10 μM against Bel-7402 cells and 1.32–6.78 μM against HepG 2 cells). At the same time, the selectivity between tumor and normal cells was observed, since low antiproliferative activity was obtained against the L-02 cell line. Besides, the anticancer potency of triterpenoid/furoxan-based NO-donating analogues against multidrug-resistant cancer cells was also developed by Zhang’s group [32,43]. Some furoxan-based NO donor/coumarin [40] and diterpenoid [44] hybrids were also developed as antitumor agents. So, the synthesis of natural product/NO donor derivatives is the feasible approach to get promising anticancer candidates.

The reasons above stimulated us to combine two of our molecules of interest together. So, a series of furoxan-based NO-releasing enmein-type *ent*-kaurane diterpenoid derivatives were developed. The antiproliferative activity of these hybrids was tested against four human cancer cell lines (Bel-7402 hepatoma, K562 leukemia, MGC-803 gastric cancer, and CaEs-17 esophageal cancer cells) and normal liver L-02 cells. The NO-releasing ability was measured by Griess assay. Then the preliminary structure activity relationship (SAR) was concluded based on these experimental data. Furthermore, the investigation of molecular mode of action and effects on apoptosis-related proteins in Bel-7402 cells was also carried out.

## 2. Introduction

### 2.1. Synthesis of Compounds ***2**–**9*** and ***10a**–**10i***

**Scheme 1:**
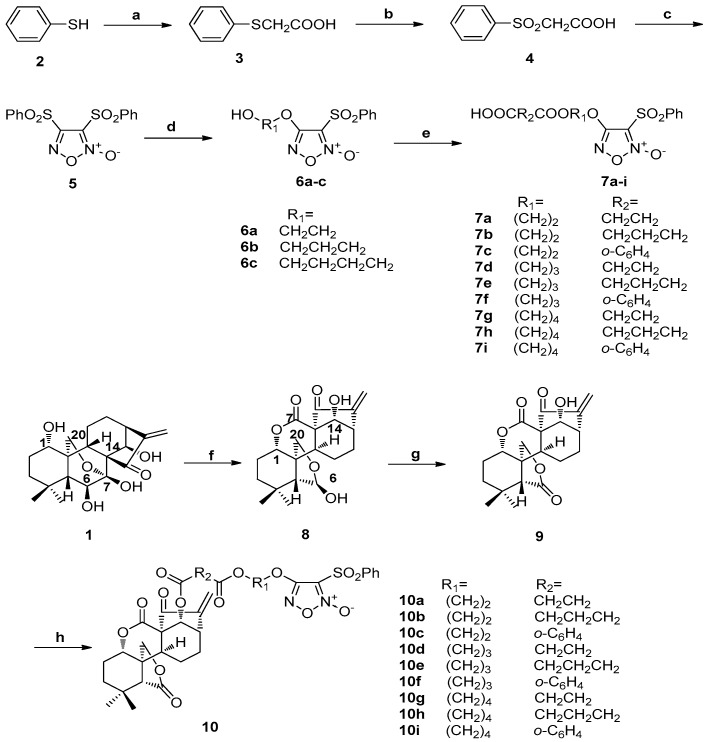
Synthesis of NO donor derivatives **10a**–**i**. *Reagents and conditions*: (**a**) chloroactic acid, NaOH (aq), 60 °C, 4 h; (**b**) 30% H_2_O_2_, glacial acetic acid, rt, 2 h; (**c**) fuming HNO_3_, glacial acetic acid, 60 °C, 4 h; (**d**) corresponding diol, NaOH (aq), THF, rt, 2 h; (**e**) anhydride, DMAP/TEA, DCM, rt, 1 h; (**f**) sodium periodate, H_2_O, rt, 6 h; (**g**) Jones reagent, DCM, rt, 0.5 h; (**h**) **7a**–**i**, EDCI, DMAP, DCM, rt, 8–12 h.

The synthesis routine is illustrated in Scheme 1. The starting material benzenethiol (**2**) was converted to diphenylsulfonylfuroxan (**5**) in a three-step sequence. Monophenylsulfonylfuroxans (**6a**–**c**) were synthesized through the reactions of **5** by treatment with corresponding diols (ethane-1,2-diol, propane-1,3-diol, and butane-1,4-diol). Finally, different anhydrides (succinic anhydride, glutaric anhydride, and phthalic anhydride) were reacted with **6a**–**c** to obtain the key intermediate furoxan-based NO donors **7a**–**i** [45]. Starting from commercially available *ent*-kaurane diterpenoid oridonin (**1**), enmein-type 6-oxo-6,7-*seco*-kaurane diterpenoid (**9**) was obtained by two steps of oxidation in total yields of 87% [10]. Compound **9** was reacted with furoxan-based NO donors **7a**–**i** to produce target hybrids **10a**–**i**.

### 2.2. Antiproliferative Activities in Vitro and SAR

The antiproliferative activity of **7a**–**i** and **10a**–**i** was evaluated using the MTT method against Bel-7402, L-02, K562, MGC-803, and CaEs-17 cell lines. The results are summarized in Table 1. Compounds **7a**–**i** exhibited stronger antiproliferative activity against four selected tumor cell lines than in normal liver cell line L-02, with IC_50_ values ranging from 16.7 to 25.7 μM. Compounds **7d**–**f** with R_1_ of (CH_2_)_3_ exhibited stronger cytotoxicity in tumor cells than those with R_1_ of (CH_2_)_2_ and (CH_2_)_4_. From these data, no obvious SAR could be concluded when R_2_ was changed among (CH_2_)_2_, (CH_2_)_3_, and *o*-C_6_H_4_. It was also found that all the target derivatives **10a**–**i** exerted stronger antiproliferative activities than the lead oridonin against all four selected tumor cells and parent compound **9** against Bel-7402, MGC-803, and CaEs-17 cancer cell lines. In K562 cells, the IC_50_ values of **10a**–**i** ranged from 1.73 to 2.90 μM, while parent compound **9** showed IC_50_ value of 2.64 μM. The target compounds **10** exhibited more potent cytotoxic activity than corresponding NO donors **7**, which was also consistent with the literature [46]. Compounds **10a**–**c** had the same R_1_ of (CH_2_)_2_, and R_2_ were (CH_2_)_2_, (CH_2_)_3_, and *o*-C_6_H_4_, respectively. Among them, **10c** with R_2_ of *o*-C_6_H_4_ showed the strongest in vitro antiproliferative activity with IC_50_ values of 1.01, 1.97, 1.60, and 4.02 μM against Bel-7402, K562, MGC-803, and CaEs-17 cell lines, respectively. Similar SAR could also be concluded from **10d**–**f** and **10g**–**i**, that R_2_ of *o*-C_6_H_4_ was favorable. In general, when R_2_ were aromatic groups *o*-C_6_H_4_ (**10c**, **10f** and **10i**), the antiproliferative potency was stronger than those of alkyl groups. Compounds **10a**, **10d**, and **10g** bearing the same R_2_ of (CH_2_)_2_ and **10d** with R_1_ of (CH_2_)_3_ exhibited smaller IC_50_ values against the four cancer cell lines. In group **10b**, **10e**, and **10h**, and group **10c**, **10f**, and **10i**, similar SAR could also be observed that **10e** and **10f** with R_1_ of (CH_2_)_3_ showed stronger cytotoxic activity.

**Figure 2:**
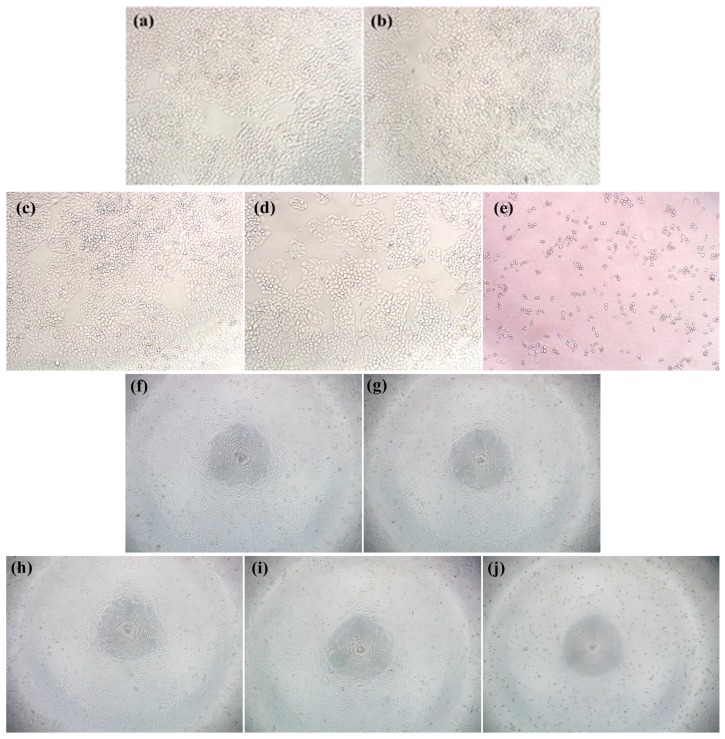
Morphology effects of **10f** on Bel-7402 (**a**–**e**, magnification: 100 times) and L-02 cells (**f**–**j**, magnification: 40 times). Cells were treated with compound **10f** for 48 h, and then observed under light microscopy. (**a**) Negative control; (**b**) solvent control (DMSO); (**c**) cells treated with 0.125 μM **10f**, (**d**) cells treated with 0.5 μM **10f**; (**e**) cells treated with 2 μM **10f**; (**f**) negative control; (**g**) solvent control (DMSO); (**h**) cells treated with 12.5 μM **10f**; (**i**) cells treated with 25 μM **10f**; (**j**) cells treated with 50 μM **10f**.

Interestingly, all the target molecules **10a**–**i** showed selective cytotoxicity between tumor and normal liver cells, with IC_50_ values ranging from 22.1 to 33.9 μM against L-02 cells. Compound **10f** with R_1_ of (CH_2_)_3_ and R_2_ of *o*-C_6_H_4_ was the most potent one against the four selected cancer cell lines. The IC_50_ values were 0.81, 1.73, 1.18, and 3.77 μM, which were 8.2-, 1.8-, 3.8-, 1.9-fold and 18.7-, 0.5-, 3.9-, 5.3-fold stronger than **1** and **9**, respectively. The morphology effects of **10f** on Bel-7402 cells are shown in Figure 2. Compound **10f** also showed a good SI (selectivity index) of 36.9, while the SI values of lead oridonin and parent compound **9** were about 2.4 and 1.6, respectively. This indicated that the NO-releasing derivatives could obviously enhance the selectivity. Based on the MTT assay, most derivatives showed remarkable antiproliferative effects against Bel-7402 cells with IC_50_ values ranging from 0.81 to 1.91 μM. Consequently, the Bel-7402 cell line was selected for intensive mechanism studies.

### 2.3. NO-Releasing Ability

**Figure 3:**
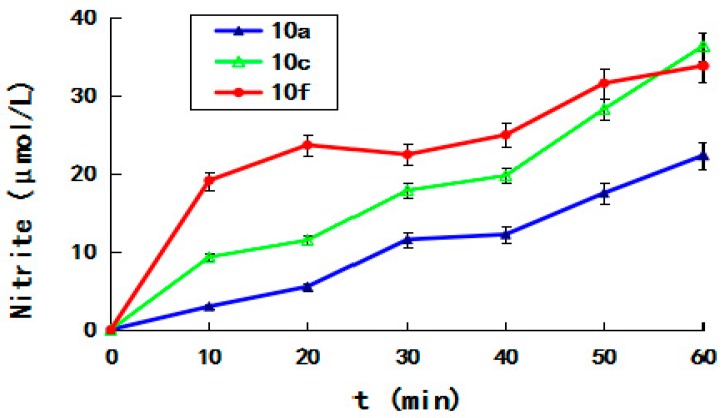
Griess assay of compounds **10a**, **10c** and **10f** measured at the time points of 0, 10, 20, 30, 40, 50, and 60 min.

Exogenous release of nitric oxide by furoxan-based NO-donating enmein-type 6,7-*seco*-*ent*-kaurane diterpenoid derivatives might contribute to the cytotoxicity. The formation of nitrite (NO^2−^) was a reliable proxy measure for nitric oxide, in the presence of thiol. Therefore, the levels of nitrate/nitrite in the lysates of the selective hybrids **10a**, **10c**, and **10f** were determined at 100 μM by Griess assay over the duration of 0–60 min and measured at the time points of 10, 20, 30, 40, 50, and 60 min. As shown in Figure 3, the concentration of NO increased with time, and at all the time points measured (10, 20, 30, 40, and 50 min) **10f** released more NO than **10a** and **10c**. At the time point of 60 min, **10a**, **10c**, and **10f** produced 22.2, 36.2, and 33.7 μmol/L of NO, respectively.

### 2.4. Effect of Cell Cycle

**Figure 4:**
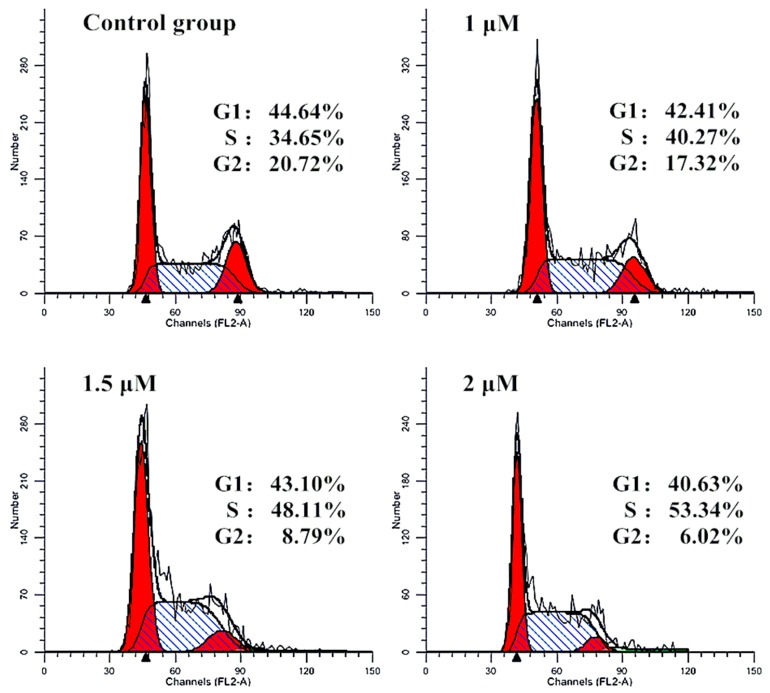
Influence of the cell cycle arrest of S phase by **10f** in Bel-7402 cells: Bel-7402 cells were incubated with the indicated concentrations of **10f** for 24 h and the cells were stained with propidium iodide (PI). Cellular DNA content, for cell cycle distribution analysis, was measured using a flow cytometer. The diagrams show the distribution of the cells according to their DNA content. The inserts give the percentages of cells in different cell cycle phases.

In general, anticancer agents prevented cell division at various checkpoints of cell cycle, thereby decreasing the growth and proliferation of cancerous cells. To determine whether the suppression of cell growth by furoxan-based NO donor/enmein-type 6-oxo-6,7-*seco*-kaurane diterpenoid hybrid **10f** was caused by cell-cycle effect, we further detected the DNA content of cell nuclei by flow cytometry (Figure 4). Bel-7402 cells were treated with **10f** at concentrations of 1.0, 1.5, and 2.0 μM, which resulted in accumulation of 40.27%, 48.11%, and 53.34% of cells at the S phase, respectively, compared with the untreated cells. There were almost no changes of G_1_ phase cells, and the decline of G_2_ phase cells were observed of 17.32%, 8.79%, and 6.02%, respectively. The influence of cell-cycle progression at low micromolar concentrations of NO donor/enmein-type diterpenoid hybrids would make these compounds promising agents for use in combination with anticancer drugs acting at different stages of the cell cycle.

### 2.5. Induction of Apoptosis

**Figure 5:**
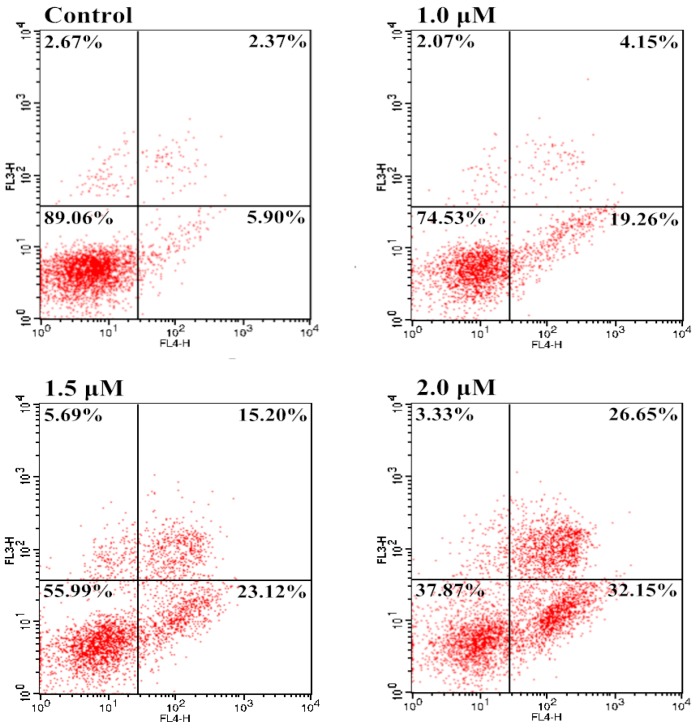
Apoptosis in Bel-7402 cells by treatment with **10f**: Bel-7402 cells were incubated with different concentrations of **10f** for 24 h and the cells were stained with annexin V-FITC and PI, followed by flow cytometry analysis.

Apoptosis is a process of programmed cell death that occurs in multicellular organisms and clears the normal aging and damaged cells. However, cancer cells usually have an abnormal ability to proliferate mainly due to defective apoptosis. Thus, activation of apoptosis can be a therapeutic method for cancer by reducing accumulation of cancer cells [47]. In order to examine the involvement of apoptosis in the loss of cancer cell viability of the most potent hybrid **10f**, an annexin V-FITC/propidium iodide (PI) binding assay was carried out. Bel-7402 cells were exposed to different concentrations of **10f** and percentages of apoptotic Bel-7402 cells were determined by flow cytometry. As shown in Figure 5, **10f** exhibited potent dose-dependent activity in the induction of apoptosis. Treatment of Bel-7402 cells with **10f** at 1.0, 1.5, and 2.0 μM, apoptotic cell rates (early and late) were 23.41%, 38.32%, and 58.80%, as compared with 8.27% in an untreated vehicle control, indicating that **10f** was able to induce apoptotic cell death in Bel-7402 cells in a concentration-dependent manner.

### 2.6. Effect of Mitochondrial Depolarization

**Figure 6:**
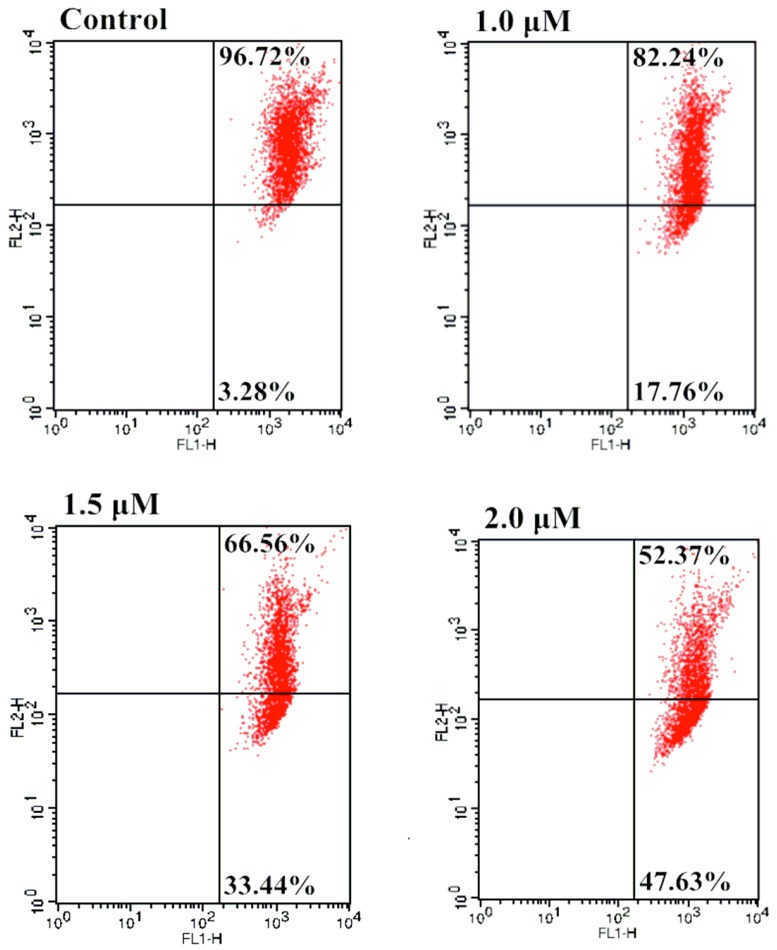
Effect of **10f** on the mitochondrial membrane potentials of Bel-7402 cells: Bel-7402 cells were incubated with the indicated concentrations of **10f** for 48 h prior to staining with JC-1.

Mitochondrial changes, including loss of mitochondrial membrane potential, were key events that took place during drug-induced apoptosis. Apoptotic signals could result in the loss of mitochondrial membrane potential in the process of apoptosis. The collapse of the mitochondrial transmembrane potential had been shown to promote mitochondrial permeability transition and induced the release of proapoptotic molecules, including apoptosis-inducing factors and cytochrome c, from the intermembrane space in the cytoplasm [48]. Subsequently, cytochrome c interacted with procaspase-9 to form the apoptosome leading to activation of caspase-9 and downstream effector caspases. The caspase-9 in turn activated the effector caspase-3 which on the other hand induced the apoptotic signaling cascade [49]. In the resent research of our group, we found cytochrome c played an important role in the lead compound oridonin **1**-induced mitochondrion-mediated apoptosis [50]. To determine whether the apoptosis induced by furoxan-based NO-releasing enmein-type *ent*-kaurane diterpenoid derivative **10f** was through mitochondrial mediated pathway, Bel-7402 cells were incubated with different concentrations (0, 0.5, 1.5, 2.0 μM) of **10f** prior to staining with the lipophilic mitochondrial probe JC-1. The number of cells with collapsed mitochondrial membrane potentials in different groups of cells was determined by flow cytometry analysis (Figure 6), yielding 3.28%, 17.76%, 33.44%, and 47.63% apoptotic cells, respectively. These results demonstrated that incubation with **10f** caused mitochondrial depolarization of Bel-7402 cells.

### 2.7. Effect of Apoptosis-Related Proteins

**Figure 7:**
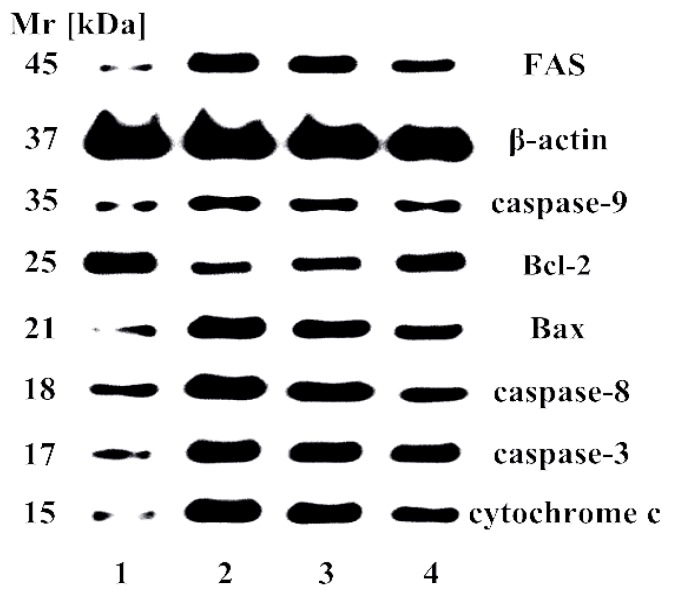
Western blotting of **10f** on the expression of apoptosis-related proteins in Bel-7402 cells. 1: negative control; 2: 2.0 μM; 3: 1.5 μM; 4: 1.0 μM.

Caspases played an important role in the apoptotic signaling network, and apoptotic pathways depend on activation of caspases for the final execution of apoptosis. Therefore, caspase activity assay was performed to determine whether caspases were responsible for the apoptotic effects of **10f** in Bel-7402 cells. Bcl-2 family proteins are important in the regulation of cell apoptosis and the pro-apoptotic protein Bax can be recruited to the mitochondrion and trigger cytochrome c release into cytoplasm to activate caspase-9 and -3 [51]. Anti-apoptotic protein Bcl-2 can inhibit the function of pro-apoptotic proteins by interaction and cancers with high levels of Bcl-2 show drug resistance in clinical cancer treatment [52]. It was found that there was upregulation of proapoptotic caspase-3, -8, -9, Bax, FAS, and cytochrome c molecules, and downregulation of anti-apoptotic Bcl-2 expression treated with **10f** for 48 h in a concentration-dependent manner (Figure 7). The molecular mode of action revealed that **10f** caused cell-cycle arrest of S phase and induced apoptosis in Bel-7402 cells through mitochondria-related caspase-dependent oxidative stress-triggered caspase 3-, 8-, and 9-dependent pathways.

## 3. Materials and Methods

### 3.1. Chemistry

#### 3.1.1. General

All reagents were obtained commercially and used without further purification. Melting points were taken on an XT-4 micro melting point apparatus and uncorrected. ^1^H-NMR spectra were recorded with a Bruker AV-300 spectrometer (Bruker Corp., Karlsruhe, Germany) in the indicated solvents (TMS as internal standard) and ^13^C-NMR spectra were recorded with a Bruker AV-400 or AV-600 spectrometer (Bruker Corp.); chemical shifts were expressed as ppm against TMS as an internal reference. Splitting patterns were designed as s, singlet; d, doublet; t, triplet; m, multiplet. Mass spectra were obtained with an FTMS-2000 instrument (Thermo Fisher Scientific, Waltham, MA, USA). HRMS data were collected with an Agilent QTOF 6520 (Agilent Technologies China, Beijing, China). The reactions were monitored by thin-layer chromatography (TLC) on glass-packed precoated silica gel GF254 plates and visualized with a UV lamp. Flash column chromatography was performed using silica gel (200–300 mesh) purchased from Qingdao Haiyang Chemical Co. Ltd (Qingdao, China).

#### 3.1.2. General Procedure to Synthesize **10**

Compound **9** (72 mg, 0.2 mmol) was dissolved in 15 mL CH_2_Cl_2_ and reacted with the corresponding NO donor **7** (0.24 mmol), in addition of EDCI (0.4 mmol) and DMAP (0.02 mmol). After stirring at room temperature for 8–12 h, the mixture was poured into 15 mL 10% HCl and extracted with CH_2_Cl_2_ (3 × 10 mL). The organic layer was combined, washed sequentially with H_2_O and saturated NaCl solution, dried over anhydrous Na_2_SO_4_, and concentrated in vacuo. The crude product was purified by column chromatography (MeOH/CH_2_Cl_2_ 1:300 *v*/*v*) to give the title compounds. The data of compounds **10a**–**e** and **10g**–**i** were in Supplementary Materials.

Compound **10f**: white solid, mp. 151–154 °C, yield 40%. ^1^H-NMR (CDCl_3_, 300 MHz), δ (ppm) 8.09 (2H, d, *J* = 7.5 Hz, Ar-H), 7.76 (2H, m, Ar-H), 7.65 (3H, m, Ar-H), 7.52 (2H, m, Ar-H), 6.24 (1H, s, 17-CH_2_), 5.86 (1H, s, 14-CH), 5.61 (1H, s, 17-CH_2_), 4.62 (1H, m, 1-CH), 4.58 (2H, m, -CH_2_), 4.47 (2H, m, -CH_2_), 4.06, 4.36 (each 1H, dd, *J*_A_ = *J*_B_ = 9.9 Hz, 20-CH_2_), 3.29 (1H, d, *J* = 9.0 Hz, 13-CH), 1.22 (3H, s, 18-CH_3_), 1.06 (3H, s, 19-CH_3_); ^13^C-NMR (CDCl_3_, 400 MHz) δ 197.37, 175.45, 167.21, 166.87, 166.09, 159.09, 147.03, 138.07, 135.87, 131.85, 131.69, 131.46, 130.94, 129.97, 129.93 (×2), 128.82, 128.73 (×2), 121.79, 110.70, 74.67, 71.51, 68.03, 61.34, 59.59, 50.90, 47.75, 46.31, 40.45, 36.40, 33.19, 32.36, 29.68, 27.97, 23.77, 23.21, 22.82, 19.19; MS (ESI) *m*/*z*: 808.3 [M + NH_4_]^+^, 790.9 [M + H]^+^, 825.1 [M + Cl]^−^; HRMS (ESI, M + NH_4_) *m*/*z* calcd for C_39_H_42_N_3_O_14_S: 808.2382, found: 808.2371.

### 3.2. Biology

#### 3.2.1. MTT Assay

Cytotoxicity assay in vitro was employed by the MTT assay following the methodology described previously, which was performed in flat-bottomed 96-well plates. In short, exponentially growing MGC-803 cells at the log phase were added to each well at a density of 5 × 10^3^ per well, then treated with serial dilutions of the compounds in three replicates at various concentrations (0.039–40 µg/mL). After 72 h, 20 µL of MTT solution (5 mg/mL) per well was added to each cultured medium. Then, DMSO was added to each well (150 µL/well). After 10 min at room temperature, the absorbance (OD) was read on a microplate reader at the wavelength of 490 nm (BIO-RAD Instruments Inc. 550, Hercules, CA, USA). In these experiments, taxol was used as the positive reference with the concentration of 10 µg/mL. The concentration causing 50% inhibition of cell growth (IC_50_) was determined. The same method was used in the test against L-02, CaEs-17, Bel-7402, and K562 cell lines.

#### 3.2.2. Griess Assay

NO-releasing ability was determined by assaying the levels of NO^2−^ using the Griess reagent. The levels of nitrate/nitrite formed from individual compounds were determined by nitrate/nitrite, in triplicate with 100 μM of individual compounds for 0–60 min according to the manufacturer’s instructions at the time points of 0, 10, 20, 30, 40, 50, and 60 min (Beyotime, Nanjing, China). The lysates were mixed with Griess reagent for 40 min and centrifugalized for 10 min, and then measured at 540 nm.

#### 3.2.3. Cell Cycle Study

Progression through the cell cycle was assessed by flow cytometry DNA determination with PI. Bel-7402 cells were plated in 6-well plates (5.0 × 10^3^ cells/well) and incubated at 37 °C for 24 h. Exponentially growing cells were then incubated with tested compound at a certain concentration in triplicate. Untreated cells (control) or cells treated with the solvent (DMSO) of the compound were included. After 48 h treatment, cells were centrifuged and fixed in 70% ethanol at 4 °C overnight and subsequently resuspended in PBS containing 100 μL RNase A and 400 μL PI. Cellular DNA content, for cell cycle distribution analysis, was measured using a flow cytometer (FACS Calibur Becton-Dickinson, Franklin Lake, NJ, USA).

#### 3.2.4. Analysis of Cellular Apoptosis

The Bel-7402 cells were seeded in 6-well plates to grow overnight, and then treated with or without **10f** at indicated concentrations in triplicate for 24 h. Cells were then washed twice in PBS and resuspended in annexin V binding buffer. Annexin V-FITC was then added and the mixture was incubated for 15 min under dark conditions at 25 °C. PI was added just prior to acquisition. Apoptosis was analyzed using annexin V and PI double-staining by flow cytometry according to the manufacturer’s instructions in order to detect apoptotic cells.

#### 3.2.5. Mitochondrial Membrane Potential Assay

Briefly, Bel-7402 cells were incubated in triplicate with the test compound **10f** (1.0, 1.5 and 2.0 μM) or vehicle for 48 h, and then washed with PBS and stained with JC-1 dye under dark conditions according to the manufacturer’s instruction (KeyGen Biotech, KGA601). The percentage of cells with healthy or collapsed mitochondrial membrane potentials was monitored by flow cytometry analysis.

#### 3.2.6. Western Blot Analysis

Bel-7402 cells were incubated in triplicate with different doses (1.0, 1.5 and 2.0 μM) of **10f** for 48 h. After the protein concentrations were determined, individual cell lysates were separated by sodium dodecyl sulfate polyacrylamide gel electrophoresis (10% gel, SDS-PAGE) and transferred onto nitrocellulose membranes. After being blocked with 5% fat-free milk, the target proteins in the membranes were probed with monoclonal anti-Bax (KGA714), anti-Bcl2 (KGA715), anti-caspase 3 (KGA717), anti-caspase 9 (KGA720), anti-cyto C (KGA723), and anti-β-actin antibodies (KGA731, KeyGEN Biotech, Nanjing, China), respectively. The relative levels of each signaling event to control β-actin were determined by densimetric scanning.

## 4. Conclusions 

In this effort, a series of NO-donating/enmein-type diterpenoid hybrids **10a**–**i** with potent antiproliferative activities was prepared. The levels of nitrate/nitrite in the cell lysates were tested by Griess assay and the results showed that the improved antiproliferative activity could be attributed to its NO releasing to some extent. The preliminary SAR of the target compounds was discussed based on the experimental data. In all the synthetic hybrids, **10f** with R_1_ of (CH_2_)_3_ and R_2_ of *o*-C_6_H_4_ was the most potential one. The IC_50_ values were 0.81, 1.73, 1.18, and 3.77 μM against Bel-7402, K562, MGC-803, and CaEs-17 human cancer cells, respectively. It also showed the best cytotoxic selectivity between tumor and normal liver L-02 cells. Furthermore, the investigation concerning the molecular mode of action revealed that **10f** caused cell cycle arrest of S phase and induced apoptosis in Bel-7402 cells through mitochondria-related caspase dependent pathways. It is expected that these kinds of NO-donor/diterpenoid hybrids could provide a promising approach for the discovery of novel antitumor agents.

## Figures and Tables

**Table 1:** The antiproliferative activity (IC_50_^a^ μM) of synthetic hybrids **10a**–**i** against selected cell lines ^b^.

Compound	Bel-7402	L-02	K562	MGC-803	CaEs-17
**1**	7.48 ± 0.32	18.3 ± 0.8	4.76 ± 0.32	5.69 ± 0.39	11.0 ± 1.0
**9**	16.0 ± 1.1	26.4 ± 1.3	2.64 ± 0.19	5.84 ± 0.57	23.7 ± 1.8
**7a**	22.0 ± 0.8	37.8 ± 1.3	22.6 ± 2.0	25.4 ± 1.5	24.3 ± 0.9
**7b**	22.3 ± 1.6	39.6 ± 1.4	24.2 ± 2.5	22.2 ± 2.5	25.7 ± 1.4
**7c**	19.6 ± 1.0	41.3 ± 2.9	22.6 ± 1.7	24.6 ± 1.4	23.8 ± 1.0
**7d**	17.8 ± 1.4	37.4 ± 0.8	17.5 ± 1.4	18.5 ± 0.8	19.8 ± 1.3
**7e**	18.6 ± 0.8	36.3 ± 2.1	18.5 ± 1.6	20.0 ± 1.1	18.7 ± 1.0
**7f**	16.7 ± 0.8	35.8 ± 1.3	17.5 ± 1.0	19.3 ± 0.6	19.2 ± 0.7
**7g**	22.7 ± 1.0	39.4 ± 2.6	24.0 ± 1.4	20.6 ± 1.7	25.7 ± 0.7
**7h**	21.3 ± 0.7	35.4 ± 1.4	24.5 ± 1.4	23.5 ± 1.0	22.9 ± 1.4
**7i**	22.4 ± 1.6	37.9 ± 2.8	23.0 ± 1.2	21.6 ± 1.5	24.8 ± 1.5
**10a**	1.91 ± 0.09	24.4 ± 0.8	2.86 ± 0.05	2.84 ± 1.12	5.68 ± 0.53
**10b**	1.75 ± 0.12	22.1 ± 1.3	2.90 ± 0.17	2.67 ± 0.24	5.56 ± 0.06
**10c**	1.01 ± 0.04	31.2 ± 1.2	1.97 ± 0.18	1.60 ± 0.15	4.02 ± 0.14
**10d**	1.17 ± 0.02	27.4 ± 1.0	2.04 ± 0.10	2.03 ± 0.15	4.84 ± 0.47
**10e**	1.12 ± 0.09	29.3 ± 0.9	2.02 ± 0.17	1.91 ± 0.09	4.04 ± 0.23
**10f**	0.81 ± 0.06	29.9 ± 1.4	1.73 ± 0.04	1.18 ± 0.10	3.77 ± 0.30
**10g**	1.85 ± 0.11	33.9 ± 1.5	2.82 ± 0.22	2.80 ± 0.09	5.52 ± 0.08
**10h**	1.65 ± 0.12	29.8 ± 0.9	2.78 ± 0.20	2.65 ± 0.16	5.48 ± 0.21
**10i**	1.09 ± 0.09	27.7 ± 1.3	2.06 ± 0.16	1.64 ± 0.12	4.13 ± 0.29
**Taxol**	1.89 ± 0.09	3.73 ± 0.17	0.41 ± 0.02	4.65 ± 0.29	0.43 ± 0.03

^a^ Results are expressed as mean ± S.D. of three independent experiments. ^b^ IC_50_: concentration that inhibits 50% of cell growth.

## Supplementary Materials

Supplementary materials of NMR spectrum of **10f** and spectrum data of other target compounds could be found at http://www.mdpi.com/1420-3049/21/9/1193/s1.

## Abbreviations

The following abbreviations are used in this manuscript:

SAR: Structure Activity Relationship
^1^H-NMR: Proton Nuclear Magnetic Resonance
TMS: Tetramethylsilane
MS: Mass Spectrometry
ESI: Electrospray Ionization
DCM: Dichloromethane
MTT: 3-(4,5-Dimethylthiazol-2-yl)-2,5-diphenyltetrazolium bromide
FBS: Fetal Bovine Serum
NT: Not Test
FITC: Fluorescein Isothiocyanate
