# Supplementary material for: NO-Releasing Enmein-Type Diterpenoid Derivatives with Selective Antiproliferative Activity and Effects on Apoptosis-Related Proteins
Source: Molecules. 2016 Sep 8;21(9):1193. doi: 10.3390/molecules21091193 (PMC6272893; doi:10.3390/molecules21091193)
Supplement: Supplementary file 1 [file molecules-21-01193-s001.pdf]

Supplementary Materials: NO Releasing Enmein-Type Diterpenoid Derivatives with Selective Antiproliferative Activity and Effects on Apoptosis-Related Proteins

Dahong Li, Xu Hu, Tong Han, Jie Liao, Wei Xiao, Shengtao Xu, Zhanlin Li, Zhenzhong Wang, Huiming Hua, and Jinyi Xu

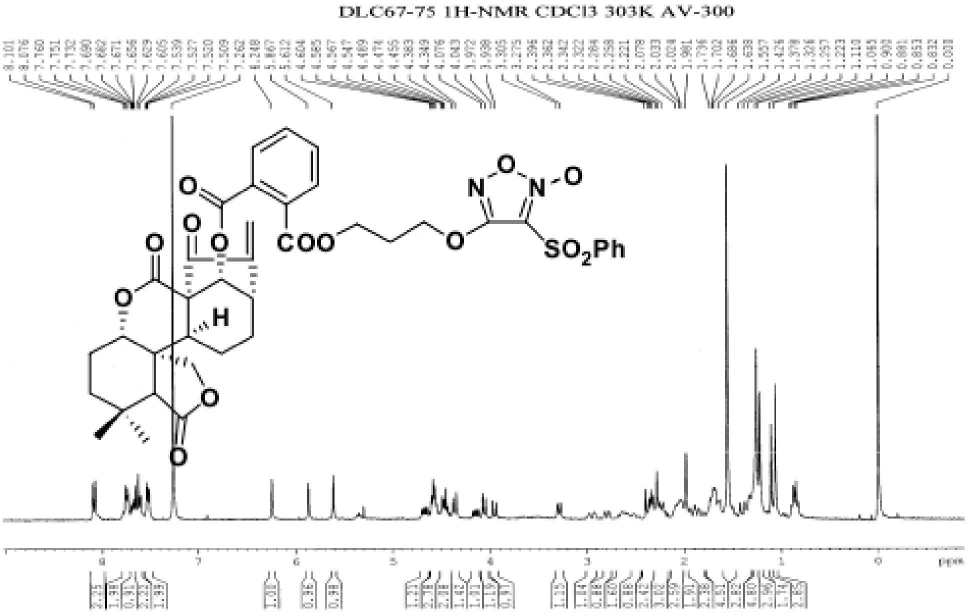

Figure S1. ^{1}H-NMR spectrum of compound **10f**.

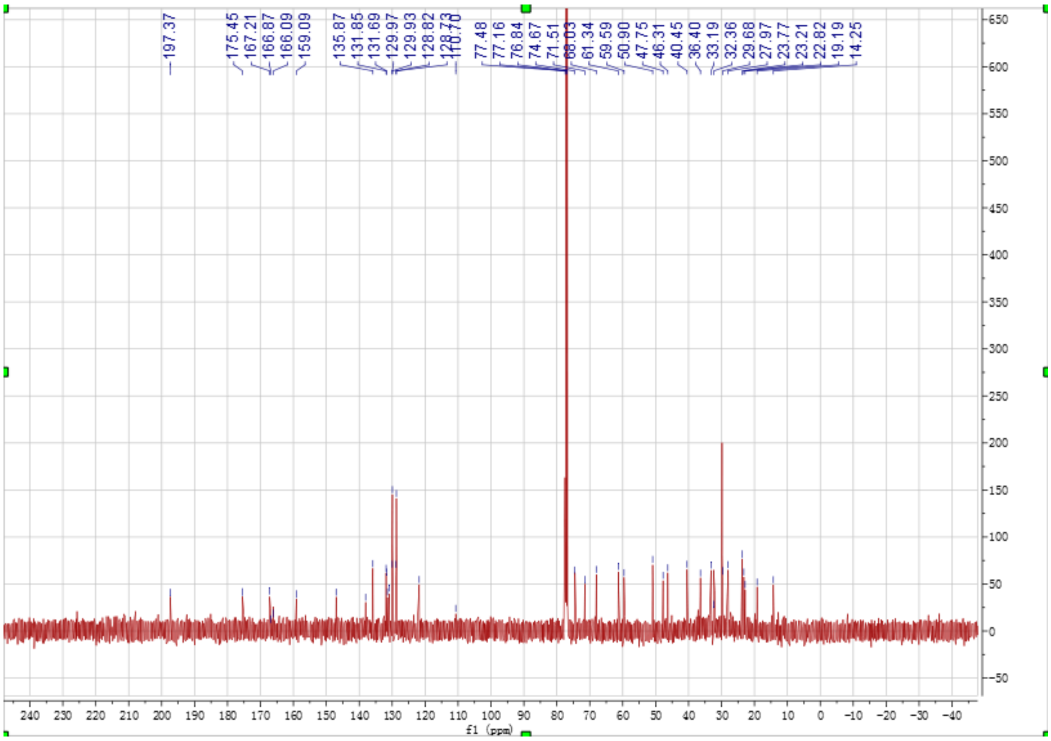

Figure S2. ^{13}C-NMR spectrum of compound **10f**.

Spectrum Data of Compounds 10a–i:

Compound **10a**: White solid, mp. 105–106 °C, yield 43%. ^{1}H-NMR ($CDCl_3$, 300 MHz), *δ* (ppm) 8.07 (2H, d, *J* = 7.5 Hz, Ar-H), 7.92 (1H, t, *J* = 7.2 Hz, Ar-H), 7.65 (2H, t, *J* = 7.5 Hz, Ar-H), 6.30 (1H, s, 17-CH_2), 5.72 (1H, s, 14-CH), 5.63 (1H, s, 17-CH_2), 4.67 (2H, m, -CH_2), 4.58 (2H, m, -CH_2), 4.46 (1H, m, 1-CH), 4.33, 4.01 (each 1H, dd, $J_A = J_B$ = 10.2 Hz, 20-CH_2), 3.19 (1H, d, *J* = 9.6 Hz, 13-CH), 2.47~2.70 (4H, m, 2×-CH_2), 2.24 (1H, s, 5-CH), 2.17 (2H, m, 12-CH_2), 1.72~1.86 (5H, m, 3,11-CH_2, 9-CH), 1.35 (2H, m, 2-CH_2), 1.20 (3H, s, 18-CH_3), 1.05 (3H, s, 19-CH_3); ^{13}C-NMR ($CDCl_3$, 400 MHz) *δ* 197.12, 175.22, 171.75, 171.63, 166.18, 158.59, 146.88, 137.83, 135.66, 129.64, 129.45, 128.48 (×2), 121.45, 110.37, 74.31, 73.62, 71.21, 68.72, 61.36, 59.37, 50.62, 47.28, 45.87, 40.09, 36.13, 32.90, 32.10, 29.38, 28.79, 28.49, 23.50, 22.86, 18.94; MS (ESI) *m*/*z*: 751.3 [M + Na]$^+$; HRMS (ESI, M + Na) *m*/*z* calcd for $C_{34}H_{36}N_2NaO_{14}S$: 751.1779, found: 751.1789.

Compound **10b**: White solid, mp. 90–91 °C, yield 46%. ^{1}H-NMR (CDCl3, 300 MHz), *δ* (ppm) 8.06 (2H, d, *J* = 7.2 Hz, Ar-H), 7.77 (1H, t, *J* = 7.5 Hz, Ar-H), 7.63 (2H, t, *J* = 7.5 Hz, Ar-H), 6.24 (1H, s, 17-CH_2), 5.70 (1H, s, 14-CH), 5.61 (1H, s, 17-CH_2), 4.61 (2H, m, -CH_2), 4.57 (1H, m, 1-CH), 4.47 (2H, m, -CH_2), 4.33, 4.02 (each 1H, dd, $J_A = J_B$ = 10.2 Hz, 20 -CH_2), 3.17 (1H, d, *J* = 9.3 Hz, 13-CH), 2.36~2.43 (4H, m, 2×-CH_2), 2.24 (1H, s, 5-CH), 2.17 (2H, m, 12-CH_2), 2.00 (2H, m, -CH_2), 1.66~1.89 (5H, m, 3,11-CH_2, 9-CH), 1.42 (2H, m, 2-CH_2), 1.05 (3H, s, -CH_3), 0.89 (3H, s, -CH_3); ^{13}C-NMR ($CDCl_3$, 600 MHz) *δ* 197.06, 175.22, 172.54, 172.45, 166.08, 158.64, 146.90, 137.99, 135.65, 129.68 (×2), 128.55 (×2), 121.50, 110.36, 74.38, 73.35, 71.28, 68.83, 61.06, 59.44, 50.74, 47.37, 45.89, 40.16, 36.32, 32.99, 32.21, 31.86, 29.47, 29.25, 27.15, 23.56, 22.62, 19.39; MS (ESI) *m*/*z*: 760.3 [M + NH_4]$^+$, 743.1 [M + H]$^+$, 777.1 [M + Cl]$^-$; HRMS (ESI, M + Na) *m*/*z* calcd for $C_{35}H_{38}N_2NaO_{14}S$: 765.1936, found: 765.1949.

Compound **10c**: White solid, mp. 118–120 °C, yield 45%. ^{1}H-NMR ($CDCl_3$, 400 MHz), *δ* (ppm) 8.01 (2H, d, *J* = 7.5 Hz, Ar-H), 7.78 (1H, m, Ar-H), 7.70 (1H, m, Ar-H), 7.61 (1H, m, Ar-H), 7.55 (2H, m, Ar-H), 7.44 (2H, t, *J* = 7.5 Hz, Ar-H), 6.21 (1H, s, 17-CH_2), 5.87 (1H, s, 14-CH), 5.58 (1H, s, 17-CH_2), 4.77 (2H, m, -CH_2), 4.72 (2H, m, -CH_2), 4.67 (1H, m, 1-CH), 4.35, 4.04 (each 1H, dd, $J_A = J_B$ = 10.2 Hz, 20-CH_2), 3.32 (1H, d, *J* = 9.6 Hz, 13-CH), 2.65 (1H, m, 5-CH), 2.24 (2H, m, 12-CH_2), 1.67~1.99 (7H, m, 2,3,11-CH_2, 9-CH), 1.05 (3H, s, 18-CH_3), 0.87 (3H, s, 19-CH_3); ^{13}C-NMR ($CDCl_3$, 400 MHz) *δ* 197.18, 175.26, 166.67, 166.37, 165.85, 158.65, 146.75, 137.73, 135.62, 131.78, 131.60, 130.92, 130.55, 129.87, 129.60 (×2), 128.77, 128.40 (×2), 121.64, 110.35, 74.44, 71.27, 68.71, 62.34, 59.33, 50.68, 47.52, 46.08, 40.20, 36.15, 32.95, 32.15, 29.60, 29.45, 23.54, 22.94, 18.93; MS (ESI) *m*/*z*: 799.3 [M + Na]$^+$; HRMS (ESI, M + Na) *m*/*z* calcd for $C_{38}H_{36}N_2NaO_{14}S$: 799.1779, found: 799.1779.

Compound **10d**: White solid, mp. 94–96 °C, yield 45%. ^{1}H-NMR ($CDCl_3$, 300 MHz), δ (ppm) 8.07 (2H, d, *J* = 7.5 Hz, Ar-H), 7.92 (1H, t, *J* = 7.2 Hz, Ar-H), 7.65 (2H, t, *J* = 7.5 Hz, Ar-H), 6.30 (1H, s, 17-CH_2), 5.72 (1H, s, 14-CH), 5.63 (1H, s, 17-CH_2), 4.67 (2H, m, -CH_2), 4.58 (2H, m, -CH_2), 4.49 (1H, m, 1-CH), 4.01, 4.33 (each 1H, dd, $J_A = J_B$ = 10.2 Hz, 20-CH_2), 3.19 (1H, d, *J* = 9.6 Hz, 13-CH), 1.19 (3H, s, 18-CH_3), 1.07 (3H, s, 19-CH_3); ^{13}C-NMR ($CDCl_3$, 400 MHz) *δ* 197.24, 175.42, 172.11, 172.11, 166.31, 159.06, 147.07, 138.12, 135.79, 129.84 (×2), 128.74 (×2), 121.70, 110.76, 74.56, 73.71, 71.44, 68.00, 60.45, 59.59, 50.90, 47.54, 46.09, 40.34, 36.42, 33.17, 32.37, 29.81, 29.64, 29.11, 28.69, 23.76, 23.13, 19.18; MS (ESI) *m*/*z*: 760.3 [M + NH_4]$^+$, 743.1 [M + H]$^+$, 777.1 [M + Cl]$^-$; HRMS (ESI, M + Na) *m*/*z* calcd for C35H38N2NaO14S: 765.1902, found: 765.1914.

Compound **10e**: White solid, mp. 98–100 °C, yield 37%. ^{1}H-NMR ($CDCl_3$, 300 MHz), *δ* (ppm) 8.06 (2H, d, *J* = 7.5 Hz, Ar-H), 7.77 (1H, t, *J* = 7.2 Hz, Ar-H), 7.63 (2H, t, *J* = 7.5 Hz, Ar-H), 6.24 (1H, s, 17-CH_2), 5.70 (1H, s, 14-CH), 5.62 (1H, s, 17-CH_2), 4.57 (1H, m, 1-CH), 4.50 (2H, t, *J* = 6.0 Hz, -CH_2), 4.25 (2H, t, *J* = 6.0 Hz, -CH_2), 4.34, 4.01 (each 1H, dd, $J_A = J_B$ = 10.2 Hz, 20-CH_2), 3.16 (1H, d, *J* = 9.6 Hz, 13-CH), 1.21 (3H, s, 18-CH_3), 1.06 (3H, s, 19-CH_3); ^{13}C-NMR ($CDCl_3$, 400 MHz) *δ* 197.23, 175.40, 172.79, 172.70, 166.23, 158.98, 147.10, 138.15, 135.81, 129.86 (×2), 128.64 (×2), 121.61, 110.59, 74.54, 73.50, 71.44, 68.13, 60.31, 59.61, 50.89, 47.55, 46.06, 40.35, 36.45, 33.16, 32.96, 32.36, 29.80, 29.63, 27.97, 23.73, 23.14, 19.67, 19.25; MS (ESI) *m*/*z*: 774.3 [M + NH_4]$^+$, 757.0 [M + H]$^+$, 791.2 [M + Cl]$^-$; HRMS (ESI, M + Na) *m*/*z* calcd for $C_{36}H_{40}N_2NaO_{14}S$: 779.2092, found: 779.2088.

Compound **10g**: White solid, mp. 82–83 °C, yield 35%. ^{1}H-NMR ($CDCl_3$, 300 MHz), *δ* (ppm) 8.06 (2H, d, *J* = 7.5 Hz, Ar-H), 7.77 (1H, t, *J* = 7.5 Hz, Ar-H), 7.64 (2H, t, *J* = 7.5 Hz, Ar-H), 6.25 (1H, s, 17-CH_2), 5.83 (1H, s, 14-CH), 5.63 (1H, s, 17-CH_2), 4.60 (1H, m, 1-CH), 4.48 (2H, t, *J* = 6.0 Hz, -CH_2), 4.34, 4.03 (each 1H, dd, $J_A = J_B$ = 10.2 Hz, 20 -CH_2), 4.21 (1H, m, -CH_2), 4.15 (1H, m, -CH_2), 3.18 (1H, d, *J* = 9.0 Hz, 13-CH), 1.22 (3H, s, 18-CH_3), 1.05 (3H, s, 19-CH_3); ^{13}C-NMR ($CDCl_3$, 400 MHz) *δ* 197.25, 175.39, 172.22, 172.16, 166.28, 159.02, 147.04, 138.13, 135.81, 129.84 (×2), 128.65 (×2), 121.70, 110.57, 74.54, 73.69, 71.42, 71.08, 64.02, 59.55, 50.85, 47.52, 46.05, 40.31, 36.39, 33.14, 32.33, 29.79, 29.11, 28.78, 25.29, 25.05, 23.72, 23.12, 19.19; MS (ESI) *m*/*z*: 774.4 [M + NH_4]$^+$, 757.2 [M + H]$^+$, 791.2 [M + Cl]$^-$; HRMS (ESI, M + Na) *m*/*z* calcd for $C_{36}H_{40}N_2NaO_{14}S$: 779.2092, found: 779.2105.

Compound **10h**: White solid, mp. 101–102 °C, yield 30%. ^{1}H-NMR ($CDCl_3$, 300 MHz), *δ* (ppm) 8.06 (2H, d, *J* = 7.2 Hz, Ar-H), 7.77 (1H, t, J = 7.5 Hz, Ar-H), 7.63 (2H, t, *J* = 7.5 Hz, Ar-H), 6.24 (1H, s, 17-CH_2), 5.70 (1H, s, 14-CH), 4.59 (1H, m, 1-CH), 4.45 (2H, t, *J* = 6.0 Hz, -CH_2), 4.34, 4.02 (each 1H, dd, $J_A = J_B$ = 10.2 Hz, 20 -CH_2), 4.15 (2H, t, *J* = 6.0 Hz, -CH_2), 3.17 (1H, d, *J* = 9.3 Hz, 13-CH), 1.21 (3H, s, 18-CH_3), 1.06 (3H, s, 19-CH_3); ^{13}C-NMR ($CDCl_3$, 400 MHz) *δ* 197.27, 175.40, 172.88, 172.69, 166.25, 159.02, 147.08, 138.12, 135.78, 129.80 (×2), 128.61 (×2), 121.68, 110.57, 74.52, 73.45, 71.41, 71.07, 63.73, 59.59, 50.85, 47.51, 46.03, 40.33, 36.39, 33.09, 32.32, 30.20, 29.59, 29.19, 25.40, 25.01, 23.70, 23.42, 23.10, 19.69; MS (ESI) *m*/*z*: 788.2 [M + NH_4]$^+$, 771.0 [M + H]$^+$, 805.1 [M + Cl]$^-$; HRMS (ESI, M + NH_4) *m*/*z* calcd for $C_{37}H_{46}N_3O_{14}S$: 788.2695, found: 788.2681.

Compound **10i**: White solid, mp. 146–148 °C, yield 53%. ^{1}H-NMR ($CDCl_3$, 300 MHz), *δ* (ppm) 8.06 (2H, d, *J* = 7.5 Hz, Ar-H), 7.73 (3H, m, Ar-H), 7.63 (2H, t, *J* = 7.8 Hz, Ar-H), 7.52 (2H, t, *J* = 3.6 Hz, Ar-H), 6.24 (1H, s, 17-CH_2), 5.95 (1H, s, 14-CH), 5.69 (1H, s, 17-CH_2), 4.66 (1H, m, 1-CH), 4.36, 3.95 (each 1H, dd, $J_A = J_B$ = 9.9 Hz, 20-CH_2), 1.11 (3H, s, 18-CH_3), 1.06 (3H, s, 19-CH_3); ^{13}C-NMR ($CDCl_3$, 100 MHz) *δ* 189.22, 177.77, 175.45, 174.26, 167.07, 156.55, 147.04, 138.12, 135.92, 131.96, 131.52, 131.43, 131.12, 129.90, 129.77 (×2), 128.93, 128.70 (×2), 121.85, 100.14, 74.51, 71.53, 71.06, 64.90, 59.58, 50.92, 47.74, 46.31, 40.40, 36.45, 33.21, 33.11, 32.39, 29.84, 29.72, 25.40, 25.11, 23.77, 19.29; MS (ESI) *m*/*z*: 822.2 [M + NH_4]$^+$, 839.0 [M + Cl]$^-$; HRMS (ESI, M + Na) *m*/*z* calcd for $C_{40}H_{40}N_2NaO_{14}S$: 827.2092, found: 827.2097.
